# Ocular MPox: A report of two cases

**DOI:** 10.1016/j.idcr.2023.e01706

**Published:** 2023-02-01

**Authors:** Devina Bhamray-Sanchez, Shyamala Subramanian, Lisa L. Dever, Debra Chew

**Affiliations:** Rutgers New Jersey Medical School, Division of Infectious Diseases, Department of Medicine

**Keywords:** Ocular Mpox, Mpox, Monkeypox, Human Monkeypox, Viral infections with ophthalmic manifestations

## Abstract

We report two cases of ocular MPox in men living with HIV, and review the clinical manifestations, diagnosis, and treatment of this rare syndrome. Our cases highlight the need for early recognition and prompt treatment for this potentially sight threatening infection.

## Introduction

Ocular MPox (formerly termed Monkeypox) is rare and has not been well-described in the 2022 outbreak of human MPox. We report two cases of ocular MPox in men living with HIV, and review the clinical manifestations, diagnosis, and treatment of this rare syndrome.

## Cases

### Patient 1

A 28-year-old man with well-controlled HIV infection on emtricitabine-rilpivirine-tenofovir alafenamide (CD4 count 951cells/mm^3^) and previously treated syphilis presented with left eye pain, redness, and decreased visual acuity for six weeks. Prior to ocular symptoms, he reported a rash on his lower back and right shoulder which resolved, and an ulcerative lesion on his penile shaft. He was evaluated for the penile lesion and treated with one dose of intramuscular benzathine penicillin for presumed primary syphilis. Six days later, he developed left eye pain. He was evaluated in the ambulatory setting and was treated with topical cyclopentolate and erythromycin and oral valacyclovir. Despite treatment, his ocular disease progressed over the following week resulting in near complete loss of vision (light perception and hand motion only), prompting admission to our hospital. The patient denied prior exposure to known contacts with MPox. He was sexually active with one male partner. He denied recent travel. Both he and his partner had not received prior vaccination for MPox.

On physical examination, the patient had left conjunctival erythema with corneal scarring and opacification (Fig. 2A). Ophthalmologic evaluation revealed visual acuity of 20/20 in the right eye. Visual acuity of the left eye was limited to hand motion. Tonometry revealed intraocular pressure of 35 mm Hg in the left eye. Slit lamp examination of the left eye revealed 2 + injection of the conjunctiva/sclera, peripheral inferior keratolysis with stromal keratitis of the cornea, and deep and quiet anterior chamber to the extent seen on exam consistent with peripheral ulcerative keratitis with superimposed stromal keratitis. Slit lamp examination of the right eye was normal.

A corneal swab was real-time polymerase chain reaction (PCR) positive for Orthopoxvirus (Opox). The patient was treated with oral tecovirimat dose 600 mg twice daily and topical ocular trifluridine. He completed a 30-day course of tecovirimat, and continued with topical trifluridine, moxifloxacin, and prednisolone acetate. One week after completion of tecovirimat, his keratitis and ulceration has healed on ophthalmic exam, but vision and photophobia had not improved.

### Patient 2

A 36-year-old man with a history of previously treated latent tuberculosis and syphilis presented with right eye pain, redness, blurry vision, photosensitivity, and watery discharge for one month.

Review of his medical records revealed that he was previously evaluated for a facial rash one week prior. Swab of a facial lesion was PCR positive for Opox. His facial lesions resolved over seven days.

Ophthalmic examination was significant for mild right eyelid edema and mild right eye conjunctivitis (Fig. 2B). The right eye had stromal interstitial keratitis and an inferior corneal ulcer. He was treated with topical moxifloxacin and fortified vancomycin and tobramycin, and oral valacyclovir without improvement. A corneal swab obtained on admission resulted PCR-positive for Opox. He was treated with oral tecovirimat and topical ocular trifluridine, tobramycin, and prednisolone. HIV therapy was initiated with bictegravir-emtricitabine-tenofovir. He completed a 30-day course of oral tecovirimat, and he continues on topical ocular trifluridine and erythromycin. Photophobia, tearing and eye pain resolved after six weeks of treatment; however, his vision did not improve. Following four weeks of treatment, corneal swab for Opox PCR test was negative.

## Discussion

**Fig. 1:**
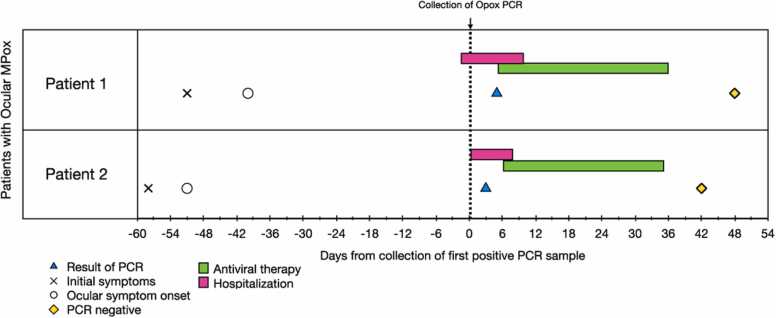
Timeline of symptoms onset, diagnostic testing, and initiation of MPox therapy for Patient 1 and Patient 2.

Ocular Mpox is a rare manifestation of acute MPox infection. In limited retrospective surveys, less than one percent of those infected with MPox developed ocular complications [1]. Our two patients highlight the devastating sight threatening complications of severe ocular MPox with keratitis. Fig. 1 illustrates a timeline of testing, symptom onset, and initiation of medical therapy for our two patients. We reviewed the literature for cases with reported ocular MPox and found 15 additional cases to date [4], [5], [6], [7], [8], [9], [10], [11], [12], [13], [14]. The spectrum of ophthalmic MPox manifestations, which include blepharitis, conjunctivitis, focal conjunctival lesions, corneal ulcerations, photophobia, keratitis, corneal scarring, and visual loss is summarized in the Table 1 [2], [3]. Mild and early complications, such as blepharitis, conjunctivitis, and focal conjunctival lesions, were usually associated with visual and symptom recovery, while late and severe complications, such as corneal ulcerations, photophobia, and keratitis, were associated with poor outcomes that included visual impairment and visual loss.

**Fig. 2:**
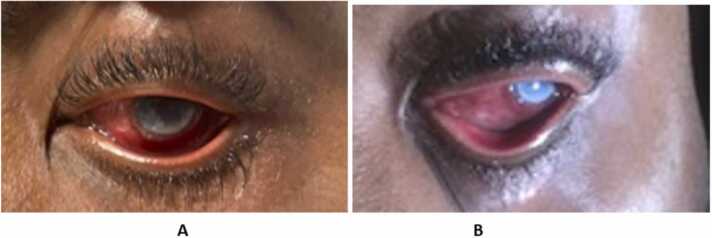
**Legend.** Ocular MPox infection manifested as unilateral conjunctivitis with corneal opacification in Patient 1 (A) and Patient 2 (**B**).

**Table 1:** Demographic and clinical characteristics, treatment, and outcomes of patients reported to have ocular MPox.

Patient	Age/Age range	Sex	HIV status (CD4 count)	Signs and symptoms	Ocular exam	MPox Treatment	Outcomes	Reference
1	28	M	Positive (951)	Ocular: eye pain, redness, and decreased visual acuity Non-ocular: rash on back, shoulder and penile shaft	Unilateral keratitis (peripheral ulcerative keratitis with superimposed stromal keratitis)	Oral TPOXX x 30 days + Topical trifluridine	Hospitalized Visual impairment	
2	36	M	Positive (200)	Ocular: eye pain, redness, blurred vision, photosensitivity, and watery discharge Non-ocular: facial lesions	Unilateral eyelid edema, conjunctivitis, corneal ulcer, interstitial keratitis	Oral TPOXX for 30 days + Topical trifluridine	Hospitalized Visual impairment	
3	35	M	Unknown	Ocular: eye pain and photophobia Non-ocular: multiple cutaneous, anogenital, and oropharyngeal lesions, generalized lymphadenopathy, and fever	Unilateral conjunctival papular lesions	IV Cidofovir (5 mg/kg) x 1 dose	Resolved	[4]
4	39	M	Unknown	Ocular: red eye and itchiness Non-ocular: chin and lip lesions	Unilateral conjunctivitis, vesicles on conjunctiva.	Unknown	Unknown	[5]
5	27	M	Unknown	Ocular: eye redness, watery discharge Non-ocular: rash on trunk, limbs, and genitalia; malaise, chills, night sweats, sore throat with difficulty swallowing	Unilateral eyelid edema, conjunctivitis, umbilicated nodules on eyelid	Oral TPOXX	Improved	[6]
6	36	F	Unknown	Ocular: eye redness and pain	Unilateral subconjunctival/upper eyelid nodules		Improved	[7]
7	42	M	Positive	Ocular: eye lacrimation, pain, and photophobia Non-ocular: rash on face and foot; fever and painful cervical adenopathy	Unilateral ulcerative lesions on eyelid margin, conjunctivitis (infiltrative lesions with conjunctival thickening)	Oral TPOXX Surgical removal of conjunctival pseudo-membranes	Resolved	[8]
8	39	M	Negative	Ocular: conjunctivitis of the left eye, with a small lesion on the lower eyelid Non-ocular: lesions back and anus, fever, back pain, cough	Unilateral blepharon-conjunctivitis, ulcerative conjunctival lesion, vesicle on lower eyelid		Resolved	[9]
9	30	M	Unknown	Ocular: photophobia, eye itchiness Non-ocular: inguinal lesions, pruritis, fever and myalgia	Unilateral edema of upper eyelid, conjunctivitis, ulcerated epithelial conjunctival lesions		Recovering	[10]
10	26	M	Unknown	Ocular: multiple eye lesions Non-ocular: papular lesions in suprapubic area; fever, malaise, headache, painful inguinal lymphadenopathy	Unilateral papular lesions in upper and lower eyelids; periorbital edema and conjunctivitis	Cidofovir (5 mg/kg) x 2 doses	Hospitalized Improved	[11]
11	38	M	Negative	Ocular: eye redness, intermittent blurred vision Non-ocular: rash anogenital area and extremities, fever, arthralgia and bilateral inguinal lymphadenopathy	Unilateral eye upper and lower lid edema, lower lid vesicle; blepharon-conjunctivitis, superficial corneal ulcers	Oral TPOXX for 2 weeks	Resolved	[12]
12	20–29	M	Positive (25)	Ocular: redness, pain, itching, swelling, discharge, foreign body sensation, photosensitivity, and vision changes Non-ocular: rash on arms, hands, chest, and buttocks	Unilateral conjunctivitis, conjunctival lesion, and keratitis	IV/PO TPOXX + Topical Trifluridine	Hospitalized Visual impairment	[13]
13	30–39	M	Positive (78)	Ocular: redness, pain, itching, and photosensitivity Non-ocular: rash on face, chest, legs, and perianal area	Unilateral medial canthus lesion, conjunctivitis, conjunctival and corneal lesion	Oral TPOXX for 24 days + Topical trifluridine	Hospitalized Improved	[13]
14	30–39	M	Negative	Ocular: redness, pain, and discharge Non-ocular: rectal pain and perianal rash	Bilateral conjunctivitis	Oral TPOXX for 30 days	Hospitalized Resolved	[13]
15	30–39	M	Negative	Ocular: redness, pain, and periorbital swelling Non-ocular: rash on abdomen, wrist, and penis	Unilateral eyelid lesion and conjunctival lesion, conjunctivitis, preseptal cellulitis	Oral TPOXX for 14 days + Topical trifluridine for 5 days IV antibiotics for preseptal cellulitis	Hospitalized Improved	[13]
16	30–39	F	Negative	Ocular: redness and pain Non-ocular: rash on vaginal labia, buttocks, back, chin, and forehead	Unilateral eyelid lesion, conjunctival and subconjunctival nodule, conjunctivitis	Oral TPOXX for 14 days + Topical trifluridine for 5 days	Hospitalized Improved	[13]
17	30	M	Negative	Ocular: conjunctival and eyelid lesions Non-ocular: rash hands, shoulder, back, penis	Unilateral eyelid edema, conjunctival and eyelid lesions, conjunctivitis	Oral TPOXX for 14 days	Resolved	[14]

Abbreviation: IV= intravenous; TPOXX = Tecovirimat; mg= milligrams; kg= kilograms

Our patients highlight the diagnostic challenges and delays and need for early recognition and prompt treatment. Ocular Mpox should be considered or suspected if there is a history of recent MPox or exposure to MPox, or prior cutaneous lesions suggestive of MPOX in patients with new ophthalmic manifestations. Most patients with ocular MPox (Table 1) had a characteristic rash with or without a preceding prodrome. The diagnosis of MPox can be confirmed by swabbing cutaneous lesions to test for nonvariola Opox by reverse transcriptase PCR testing. Acute ocular MPox is confirmed by the presence of Opox on PCR testing with conjunctival or corneal swabs [15].

Underlying host immunosuppression, such as advanced or untreated HIV infection, is associated with more severe or prolonged MPox, and may also have contributed to worse outcomes and prolonged infection in patient 2. A recent report found that among persons with MPox infections, hospitalizations were more common among persons with HIV infection than persons without HIV [16], [17]. Early initiation of antiretroviral therapy should be part of the recommended treatment in patients with advanced or untreated HIV infection with severe MPox.

Early collaboration with ophthalmology and infectious disease specialists is critical in the evaluation and treatment of patients with suspected ocular MPox. Patients should undergo urgent ophthalmic evaluation, with assessment of involved ocular structures. In addition to diagnosis and treatment for ocular MPox, patients should be assessed for bacterial, viral or fungal co-infections including *Staphylococcus aureus, Pseudomonas aeruginosa* and other bacterial pathogens, herpes simplex virus, varicella zoster virus, and syphilis.

Ocular MPox should be treated with systemic antiviral therapy. Tecovirimat (TPOXX) is the most common systemic antiviral used for severe MPox infections and is available under the Centers for Disease Control and Prevention (CDC)’s expanded access-investigational new drug protocol (EA-IND) [18], [19]. There is currently no pharmacokinetic data on the level of penetration of TPOXX on the surface or the deeper structures of the eye [18]. Moreover, there is limited data on the treatment efficacy of TPOXX, though an NIH-sponsored randomized clinical trial is currently underway to address this [20]. Currently there is no guidance on the duration of treatment for ocular MPox, though most infectious disease experts recommend treating ocular MPox with more prolonged courses of TPOXX with a minimum of 30 days depending on clinical response as opposed to a standard 14-day treatment course. The addition of Vaccina Immune Globulin Intravenous (VIGIV), may be considered for those not improving or progressing on TPOXX, in consultation with CDC [18], [21]. Finally, topical treatment with trifluridine, a topical antiviral used for the treatment of HSV keratitis, is often used in cases of conjunctivitis and keratitis associated with MPox, in consultation with an ophthalmologist. [18], [22]. Topical or oral antibiotics are frequently used in combination either to treat bacterial superinfection or as prophylactic therapy. The use of topical corticosteroids to control inflammation is controversial as it may contribute to virus persistence, and should be used in consultation with ophthalmology [21], [23].

Counseling patients with Mpox to avoid inadvertent autoinoculation of the virus into the eye, the likely route of entry of the virus, is an important preventative strategy. Patients should be counseled on hand hygiene, and on avoidance of touching their eyes, including refraining from using contact lens [13], [18], [23].

There is currently no data on the duration of infectiousness in cases with ocular MPox, but persistence of positive Opox PCR swabs in our case illustrate that this may be prolonged, and infection control precautions for patients with MPox, which include isolation, hand hygiene, and environmental disinfection, should be continued [24]. Early diagnosis and prompt treatment for patients suspected of having ocular MPox may help prevent sight threatening complications and improve patient outcomes.

## Ethical approval

This study was approved by Rutgers New Jersey Medical School.

## Funding

This research did not receive any specific grant from funding agencies in the public, commercial, or non-for-profit sectors.

## Author contributions

Devina Bhamray-Sanchez, MD: Care of patient, writing of manuscript. Shyamala Subramanian: Care of patient, writing of manuscript. Lisa L. Dever, MD: Care of patient, critical review of manuscript. Debra Chew, MD, MHP: Care of patient, writing and critical review of manuscript.

## Conflicts of Interest

The authors report no conflicts of interest.

## Consent

Patients’ written consent was obtained and a copy of the written consent is available for review by the Editor-in-Chief of this journal on request.

## Patient consent statement

Patients’ written consent was obtained for their photographs. This study was approved by Rutgers New Jersey Medical School.
